# Marine fungi of the Baltic Sea

**DOI:** 10.1080/21501203.2020.1729886

**Published:** 2020-03-12

**Authors:** Sanja Tibell, Leif Tibell, Ka-Lai Pang, Mark Calabon, E. B. Gareth Jones

**Affiliations:** a Systematic Biology, Department of Organismal Biology, Evolutionary Biology Centre, Uppsala University, Uppsala, Sweden; b Institute of Marine Biology and Centre of Excellence for the Oceans, National Taiwan Ocean University, Keelung, Taiwan; c Center of Excellence in Fungal Research, Mae Fah Luang University, Chiang Rai, Thailand; d Department of Botany and Microbiology, College of Science, King Saud University, Riyadh, Kingdom of Saudi Arabia

**Keywords:** Brackish water, diversity, Gotland, Sweden

## Abstract

Vast parts of the Baltic Sea have been mycologically neglected and are still awaiting exploration. Here we summarise earlier records of marine fungi from the Baltic, supplementing them with discoveries from fieldwork in Sweden in 2019. Although marine fungal diversity is clearly attenuated in the brackish water of the Baltic Sea, a substantial number has still been discovered. Here we list 77 species from the Baltic Sea, whereas after a critical assessment a further 18 species have been excluded as records of marine fungi. The species have mainly been identified by their morphological features, supplemented by DNA-based diagnostics. Most of the species have their main distributions in temperate areas of the Atlantic Ocean. Some of the Baltic species discovered here represent far disjunctions to tropical waters while only a very few are until now only recorded for the Baltic Sea. In this paper two species belong in Basidiomycota, while the most ascomyceteous speciose classes are Sordariomycetes (with 42 species) and Dothideomycetes (24). Halosphaeriaceae is the most speciose family in marine habitats, as also in the Baltic Sea, represented here by 29 species. Three species are new to Europe, and in addition 13 to the Baltic Sea and 13 to Sweden.

## Introduction

The Baltic Sea covers an area of 405,000 km^2^, an area slightly larger than Germany and smaller than Sweden (Bernes 2005). It constitutes one of our planet’s largest bodies of brackish water, second only to the somewhat larger Black Sea (436,400 km^2^; https://www.ceoe.udel.edu/blacksea/geography/index.html). By contrast, however, the Black Sea is on average much deeper and has a much larger volume 550,000 km^3^ versus 20,840 km^3^ for the Baltic. Both seas receive a strong influx of freshwater and also display a great variation in surface water salinity. Salinity in both seas fluctuates seasonally, between years and in longer cycles, depending on irregular influx of salt water.

**Figure 1.**
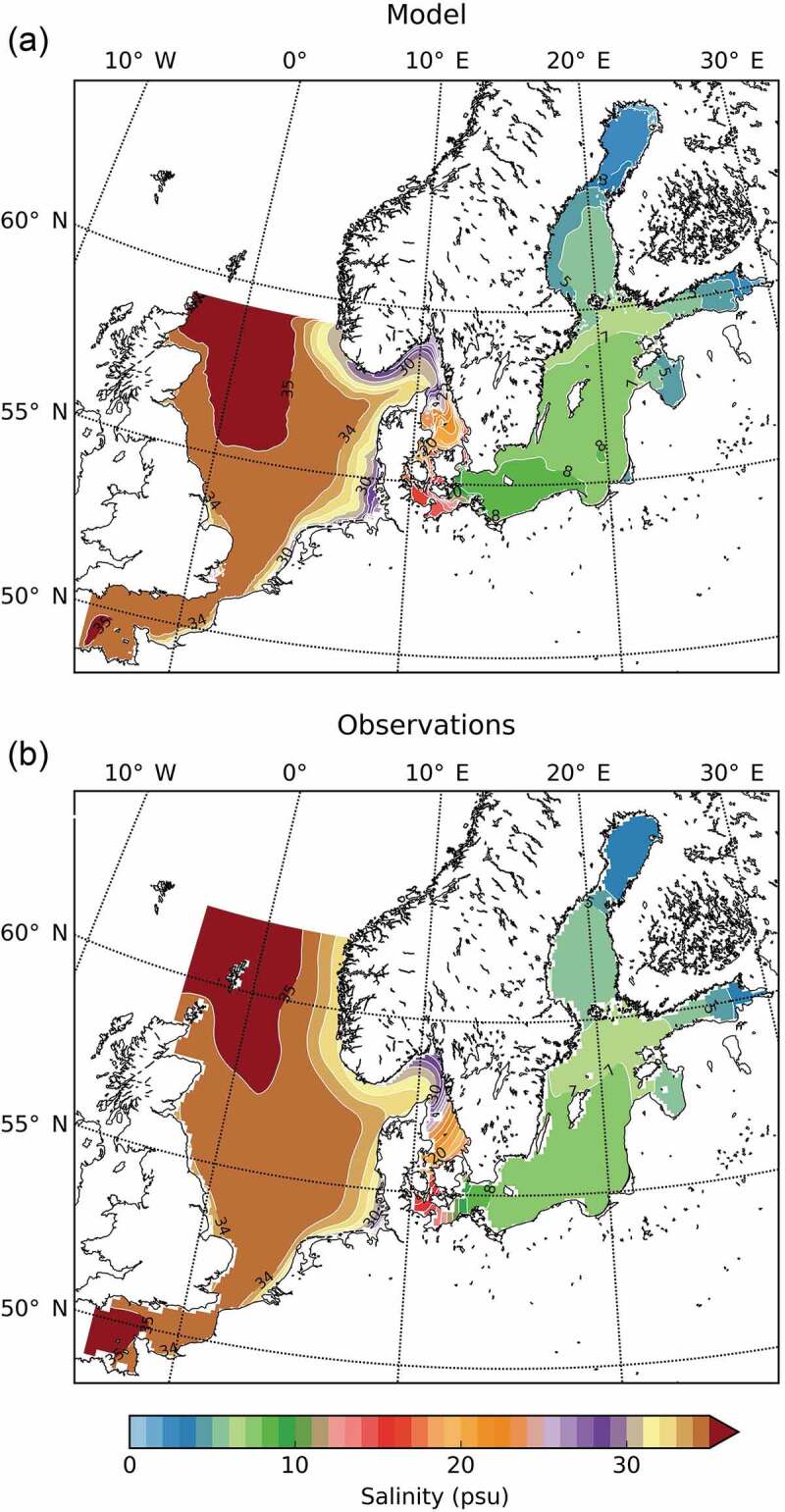
Mean surface (0–10 m) salinity for the Baltic (including North Sea and English Channel), (a) as simulated by Nemo-Nordic for the period 1979–2010 and (b) from observations (from Hordoir et al. 2019, see also Janssen et al. 1999; https://www.geosci-model-dev.net/12/363/2019/gmd-12-363-2019-f04-high-res.jpg).

In the Baltic Sea, surface salinity varies between c. 3 ‰ in the northernmost parts to some 20 ‰ close to the Danish straits and Öresund (Figure 1; from Hordoir et al. 2019; Janssen et al. 1999). The salinity of the subsurface water is generally higher and this layer is kept isolated from the surface water by a halocline, although occasional weather- and season-induced rotations of the waterbodies occurs (Bernes 2005).

The Baltic is geologically a young sea that has vacillated between being a large freshwater lake, a moderately high salinity sea, and the present state as a big body of brackish water (Bernes 2005). Consequently, the colonisation of many marine organisms into the Baltic might be comparatively recent, since the latest lake-phase ending only some 8,000 years ago. The ending of the *Littorina* Sea, some 3,000 years ago also brought a reduction in salinity to the present phase of the Baltic, called the *Limnea* Sea, as a consequence of reduced influx of ocean water with the land lifting process making the sounds to the Kattegatt shallower. For colonisation of the Baltic Sea, aquatic organisms needed to adapt to a different salinity regimen insofar that freshwater organisms had to tolerate an increasing salinity level and species adapted to ocean salinity faced a decreasing salinity in the Baltic.

The Baltic Sea has a rather low biodiversity as compared to Kattegatt or Skagerack, and the ecosystems are fragile and under pressure from eutrophication and other environmental disturbances (Bernes 2005), which i.a. has led to the development of large areas of anoxic bottoms. The Baltic Sea ecosystems and associated ecological trends have been thoroughly studied, although the emphasis has been on animal and plant communities along with studies of the algal and cyanobacterial blooms that heavily affect Baltic Sea ecology almost every summer (Bernes 2005). The precarious situation for many fish populations and lately also on the occurrences and effects of invasive species have been other major concerns.

### Tools for assessing marine fungal diversity

Recently, the diversity of cultured and observed marine planktonic fungi from across the world was summarised in a review by Hassett et al. (2019), where it was noted that the Baltic and the Mediterranean Seas have the highest fungal diversity of all areas explored. Not surprisingly, the review revealed that only half of the known marine fungal species have an available DNA locus in public databases, a circumstance that is likely to be an obstacle to accurate high-throughput sequencing classification in the future, as it certainly is already at present. Naming and describing new taxa, sequencing DNA loci/genes, has a fundamental role in marine environmental research. For marine fungi, the webpage http://www.marinefungi.org provides a search facility to genera of marine fungi, key to species and illustrations, updated information on such aspects as classification, full species descriptions, and listing of recent publications (Jones et al. 2019b). Such databases serve the scientific community in exploiting the diversity and quality of marine resources and to further research on marine fungi. In addition, as high-throughput sequencing data become more in use and available, expanding the collection of reference loci/genes and genomes will be fundamental in understanding the ecology of marine fungi.

### Baltic marine mycology

The occurrence of marine fungi in the Baltic was discovered quite late and dates back to the late 19th century (Rostrup 1884, 1888). In the wake of an increased awareness of the ecological importance and taxonomic diversity of marine fungi, more detailed investigations of the marine fungi of the Baltic Sea were undertaken during the 20th century. Höhnk (1956a, 1956b) studies the aquatic fungi from the Baltic Sea, and although mainly targeting various ecological questions (i.a. chartering salinity tolerance), a number of filamentous species were recorded in his papers, of which some were described as new. For many of the records (e.g. in Höhnk 1955), it is not possible to trace the precise locality and often materials from both the Baltic and the Atlantic were dealt with in the same paper. The thesis of Schmidt (1974a) summarised the results of a comprehensive inventory of the marine fungi of the Baltic coastline of Mecklenburg (Germany). Parts of this work had been published earlier (Schmidt 1967, 1969a, 1969b). During her research Schmidt described several new taxa that subsequently have been accepted and often found to have a wider distribution outside of the Baltic Sea. Schmidt (1974b) further investigated the distribution of marine fungi along part of the Baltic coast and noted patterns of distribution in relation to salinity zones and suggested distinctions between obligate and facultative marine species. Initially, exploration of low-salinity tolerance was surveyed when little was known about the total distribution of marine fungi (Höhnk 1956b; Schmidt 1974b). Now this knowledge is much more comprehensive and we will here try to give attention to the world-wide distribution of the species found in the Baltic Sea. There has been much nomenclatural turbulence for the marine fungi, particularly since molecular data have assumed an increasingly important part in clarifying their phylogeny and the ensuing revisions of their classification and nomenclature (Jones et al. 2019). Here we have tried to accustom the names to the more recent findings.

The aim of this paper is to update knowledge on the occurrence of marine fungal species in the Baltic. The treatment concentrates on ascomycetous and basidiomycetous filamentous fungi, thus leaving other ecologically very important groups of fungi, like Chytridiomycota and yeasts, unaccounted for. The species included are those that in some respect seem to have a consistent association with saline water. They are often found on driftwood, on macroalgae, and on shore plants – living or dead. Several have been recovered (particularly by Schmidt 1974a) on bait panels or wooden constructions along the shore-line. These habitats have been described as “marine wood” below.

## Material and methods

New and additional records in this paper originate from eight localities on Gotland, one in Södermanland, and two in Uppland in Sweden (for provinces see http://mapsof.net/sweden/provinces-ofsweden). This material was collected from driftwood and shore plant remains. Substrates detailed in the list of species are based on Baltic material.

Cultures were obtained by squashing fungal fruiting bodies from wood in seawater and spreading onto Potato Dextrose Agar (Sigma-Aldrich, USA) plates with seawater amended with Kanamycin A. The mycelial cultures obtained after incubation for 4–16 weeks were used for DNA isolation. The DNeasy Plant Mini Kit (Qiagen, Hillden, Germany) was used for isolating total DNA following the instructions of the manufacturer. Diluted (1:10) or undiluted DNA (3 μl) was used for PCR amplifications which also included the AccuPower® PCR PreMix (Bioneer, Daejeon, Republic of Korea), by adding 1.5 μl of each primer (10 μm) and water to a total volume of 20 μl. Primers used to amplify the ITS locus: ITS1 f (Gardes and Bruns 1993) and ITS 4 (White et al. 1990). Thermal cycling parameters were: initial denaturation for 4 min at 95°C, followed by 35 cycles of 1 min at 94°C, 1 min at 54°C, 45 s at 72°C, and final elongation for 5 min at 72°C. Amplification products were visualised on 0.5% agarose gels stained with gel red and the PCR product was purified using the Illustra™ ExoStar buffer diluted 10×, following the manufacturer’s protocol. Sequencing, automated reaction clean up, and visualisation were carried out as described by Macrogen Inc., Korea (www.macrogen.com). The UNITE database was queried with generated ITS sequences to infer taxonomy through molecular identification (https://unite.ut.ee/; Nilsson et al. 2018). Newly produced sequences have been deposited in GenBank.

## Results

### Diversity and ecology of Baltic marine fungi

In the following, historical records of marine fungi from the Baltic are summarised but have been included only when the locality has been unequivocally identified. Recent discoveries result from our field-work on Gotland (E.B.G. Jones, S. Tibell, and L. Tibell) and in the Swedish provinces of Södermanland and Uppland (K.-L. Pang, S. Tibell, and L. Tibell) in 2019. The distributions of the species in the Baltic Sea in the following list have been described under “Distribution”, whereas comments on the total distribution of the species sometimes has been included under “Notes”.

**1. *Amarenomyces ammophilae* (Lasch) O.E. Erikss.**

**Substrate**: Marine wood.

**Distribution**: Germany. Mecklenburg-Vorpommern, Gross-Schwansee (Schmidt 1974a, as *Camarosporium metableticum*).

*Note*. Recorded by Schmidt (1974a) as an obligate marine species occurring in the mesohaline zone. Also recorded from temperate coastal areas of the Atlantic and Pacific Oceans. A species frequently collected on *Ammophila arenaria*.

**2. *Amylocarpus encephaloides* Curr.**

**Substrate**: Marine wood.

**Distribution**: Denmark. Sjaelland. Sweden. Ångermanland, Öland, Västerbotten.

**New record**: Sweden. Gotland, Ardre par., Folhammar, 57°20ʹ45.03”N, 18°43ʹ59.11”E.

*Note*. Reported from Denmark by Rostrup (1884, 1888), Rees et al. (1979) made 64 collections on wood associated with sand in N. Jutland, Koch and Jones (1983) on wood from Nakkehoved fyr, and from Sweden by Henningsson (1974: Ångermanland, Öland) and Eriksson (2014: Västerbotten). A halophilous species, known from many areas in temperate to cold areas of the Atlantic and Pacific Oceans, particularly from localities influenced by freshwater (Koch 1974). **New** to Gotland.

**3. *Appendichordella amicta* (Kohlm.) R.G. Johnson, E.B.G. Jones, and S.T. Moss**

**Substrate**: Marine wood.

**Distribution**: Germany. Mecklenburg-Vorpommern, Gr. Jasmunder Brodden, Karlshagen (Schmidt 1974, as *Sphaerulina amicta*).

*Note*. Originally described from the French Atlantic coast it has also been reported from Chile (http://sweetgum.nybg.org/images3/2253/848/02946655.jpg as *Haligena amicta*). Also, one collection from Denmark, N Jutland (Rees et al. 1979). Here considered an oceanic species.

**4. *Arenariomyces trifurcatus* Höhnk** (Figure 3(d))

**Substrate**: Marine wood.

**Distribution**: Germany. Mecklenburg-Vorpommern, Ahlbeck, Binzer Bucht, Darss, Hiddensee, Glower Bucht, Glower Bucht, Gr. Jasmunder Brodden, Karlshagen, Gross Schwansee, Lohme, Stralsund, Warnemünde, Wissower Klinken (Schmidt 1974b, as *Corollospora trifurcata*).

**New record**: Sweden. Södermanland, Trosa par., Askö, Storsand, 58°48′27″N, 17°41′07″E.

*Note*. An oceanic species with a distribution in tropical to temperate areas of the Atlantic, Indian, and Pacific Oceans (Kohlmeyer and Kohlmeyer 1979). **New** to the East Coast of Sweden and **new** to Södermanland.

**5. *Asteromyces cruciatus* C. Moreau and Moreau ex Hennebert**

**Substrate**: Decaying *Fucus vesiculosus* and *Laminaria saccharina*. An arenicolous species frequently isolated from sand and wood associated with sand.

**Distribution**: Germany. Mecklenburg-Vorpommern, Gross-Schwansee (Schmidt 1974b, as *Camarosporium metableticum*).

*Note*. Recorded by Schmidt (1974a) as an obligate marine species occurring in the mesohaline zone. Also known from Denmark, N Jutland, isolated from wood from surface layer to a depth of 30 mm (total isolations 23, Rees et al. 1979) and many collections from sites in Jutland (Koch and Jones 1983). An oceanic species with a distribution in tropical to temperate areas of the Atlantic, Indian, and Pacific. A halophilous species.

**6. *Calycina marina* (Boyd) Rämä and Baral**

**Substrate**: *Fucus*.

**Distribution**: Sweden. Gotland (Eriksson 2014 as *Orbilia marina*; Tibell 2016), Skåne (Eriksson 2014, as *Orbilia marina*).

*Note*. Originally described from Scotland, this saprobe on *Fucus* and *Laminaria* spp. occurs in temperate parts of the Atlantic, and was i.a. reported by Baral and Rämä (2015) from Portsmouth, UK. A halophilous species.

**7. *Carbosphaerella leptosphaerioides* Schmidt**

**Substrate**: Marine wood.

**Distribution**: Germany. Mecklenburg-Vorpommern, Rügen, Glowe (Schmidt 1967).

*Note*. Originally described from water of rather low salinity in the Baltic Sea, this species has also been reported from Hawaii. Also known from Denmark, Vigsǿ (Koch and Jones 1983); 19 collections from wood in association with sand at Blokhus, and Lǿkken, Jutland (Rees and Jones 1985). A common species at various world locations. A halophilous species.

**8. *Carbosphaerella pleosporoides* I. Schmidt**

**Substrate**: Marine wood.

**Distribution**: Germany. Mecklenburg-Vorpommern, Rügen, Binzer Bucht (Schmidt 1967).

*Note*. This species was described from water of rather low salinity in the Baltic, and no additional records have been published. Jones EBG has collected it twice at Grǿnhǿj, Jutland, Denmark and considered it to be a rare species on wood associated with sand (unpublished). A halophilous species.

**9. *Ceriosporopsis halima* Linder**

**Substrate**: Marine wood.

**Distribution**: Denmark. Sjaelland, Copenhagen, Charlottenlund (Höhnk 1955, as *C. barbata*). Germany. Mecklenburg-Vorpommern, Barther Oie, Binzer Bucht, Bock, Stralsund, Prerow (Schmidt 1967, 1974a, 1974b). Sweden. Öland, Uppland (Henningsson 1974).

*Note*. Recorded by Schmidt (1974b) as an obligate marine species occurring in the mesohaline zone. Also known from Denmark, two collections from wood in association with sand at Blokhus, and Lǿkken, Jutland (Rees and Jones 1985). Originally described from the USA, it has been shown to have a distribution in coastal areas of both the western and eastern North Atlantic. It has also been recorded from the Indian and the Pacific Oceans. An early coloniser of wood submerged in the sea.

**10. *Cirrenalia macrocephala* (Kohlm.) Meyers and R.T. Moore**

**Substrate**: Marine wood.

**Distribution**: Germany, Mecklenburg-Vorpommern, several localities (Schmidt 1967, 1974a, 1974b).

*Note*. Recorded by Schmidt (1974a, 1974b)) as an obligate marine species occurring in the meso- to oligohaline zones. It is distributed in temperate to warm coastal waters of the eastern and western North Atlantic and the eastern Pacific north of the equator. Collected by Jones and Koch in Vedbaek, Denmark and at Blokhus, and Lǿkken, Jutland (Rees and Jones 1985). A halophilous species.

**11. *Coniochaeta marina* Dayarathne, S. Tibell, Tibell, and K.D. Hyde**

**Distribution**: Sweden, Bohuslän and Uppland.

**Substrate**: Driftwood.

**New records**: Sweden. Gotland, Fleringe par., 2 km NWE of Kapellshamn, Kapellshamnsviken, 57°52ʹ4.36”N, 18°48ʹ54.43”E, ST19-124 (UPS); Näs par., Nisseviken, 57°07′54″N, 18°13′02″E ST19-59b (UPS). Södermanland, Trosa par., Askö, Storsand, 58°48′27″N, 17°41′07″E, ST19-72 (UPS).

*Note*. Recently described from Bohuslän (Jones et al. 2019) and also known from Uppland. A halophilous species. **New** to Gotland and Södermanland. So far only known from the Baltic Sea and the Swedish West Coast. A collection from Sweden, Bohuslän. Skaftö par., Fiskebäckskil, Rödbergsviken, 58º15´08´´; 11º27´58´´, was collected on a large piece of driftwood by L. Tibell, June 2017, GJ 396 (MFLU 19-1240). Two recently described *Conichaeta* species have been introduced from marine habitats: *C. krabiensis* on wood from Thailand, and *C. arenariae* on *Ammophila arenaria* in Wales (Dayarathne et al. 2020).

**12. *Corollospora gracilis* Nakagiri and Tokura (**Figure 3(b))

**Substrate**: Driftwood.

**New record**: Sweden. Södermanland, Trosa par., Askö, Storsand, on sandgrains among *Zostera*, 58°48′27″N, 17°41′07″E, ST19-98 (UPS).

*Note*. This oceanic species was originally described from Japan and has also been recorded from the Eastern and Western Pacific Oceans. **New** to the Baltic Sea and Sweden.

**13. *Corollospora intermedia* I. Schmidt**

**Substrate**: Decaying thalli of *Fucus vesiculosus*.

**Distribution**: Germany, Mecklenburg-Vorpommern, see Schmidt (1969b).

*Note*. Described from the Baltic and recorded by Schmidt (1974b) as an obligate marine species occurring in the mesohaline zone. It has further been reported from Denmark by Rees et al. (1979) (Smidstrup) and Koch and Jones (1983) (Vedbaek), UK and the Mediterranean.

**14. *Corollospora luteola* Nakagiri and Tubaki**

**Substrate**: Driftwood.

**New records**: Sweden. Gotland, Fårö par., Sudersand, 57°57ʹ8.06”N, 19°15ʹ24.69”E, GJ649, GJ656 (Hb. EBG).

*Note*. Described from Japan. **New** to Europe, the Baltic Sea, and Sweden. A halophilous species.

**15. *Corollospora maritima* Werderm.**

**Substrate**: Driftwood, remains of *Fucus vesiculosus, Ceramium,* and *Zostera* spp.

**Distribution**: Germany, Mecklenburg-Vorpommern, Binzer Bucht, Gr. Jasmunder Brodden, Stralsund, Strelasund (Schmidt 1967); Ahlbeck, Graal-Müritz, Karlshagen, Kloster, Stralsund, Wissower Klinken, Gross-Schwansee (Schmidt 1974a, 1974b). Sweden. Skåne, Klagshamn (Henningsson 1974).

**New records**: Sweden. Gotland, Fårö par., Sudersand, 57°57ʹ8.06”N, 19°15ʹ24.69”E, ST19-123 (UPS); Sudersand, 57°57ʹ8.06”N, 19°15ʹ24.69”E, GJ649 (Hb. EBG). Näs par., Nisseviken, 57°07′54″N, 18°13′02″E, ST19-82 (UPS).

*Note*. Recorded by Schmidt (1974b) as an obligate marine species occurring in the mesohaline zone. An oceanic species with a distribution in the Atlantic and Pacific Oceans. Probably the most frequently reported marine ascomycete with 99 collections from Blokhus, and Lǿkken, Jutland, Denmark (Rees and Jones 1985). **New** to Gotland.

**16. *Corollospora pulchella* Kohlm., I. Schmidt and N.B. Nair**

**Substrate**: Remains of *Fucus vesiculosus*.

**Distribution**: Germany, Mecklenburg-Vorpommern, Strelasund (Kohlmeyer et al. 1967); Altefähr, Grosse Schwansee, Lohme, Wissower Klinken (Schmidt 1974b).

*Note*. Originally described from India and recorded from the Baltic Sea by Schmidt (1974b) as an obligate marine species occurring in the mesohaline zone. Also recorded from Denmark by Koch (1974) and Koch and Jones (1983). An oceanic species with a distribution in temperate to tropical areas that has subsequently also been recorded from the southwestern North Atlantic and the Pacific Oceans.

**17. *Corollospora quinqueseptata* Nakagiri (**Figure 3(a))

**Substrate**: Driftwood, sand grains.

**Distribution**: Sweden. Södermanland, Trosa par., Askö, Storsand, 58°48′27″N, 17°41′07″E.

*Note*. Originally described from Japan, and hence also reported from Florida. **New** to the Baltic Sea, and to Sweden.

**18. *Crinigera maritima* I. Schmidt**

**Substrate**: Decaying thalli of *Fucus vesiculosus* and marine wood.

**Distribution**: Germany, Mecklenburg-Vorpommern, Binzer Bucht, Glowe, Gross-Schwansee (Schmidt 1974).

*Note*. Described from the Baltic Sea, *Crinigera maritima* was introduced by Schmidt on *Fucus vesiculosus* and marine wood but upon reinvestigation of the type material it was found to comprise two separate fungi (Koch and Jones 1989). The material on wood was designated a new genus and species *Dryosphaera* (*D. navigans*) and the material on the seaweed referred to *Crinigera maritima*. Recorded by Schmidt (1974) as an obligate marine species occurring in the mesohaline zone. *Crinigera maritima* specimens collected prior to 1989 need to be sequenced, re-evaluated, and correctly identified.

**19. *Cumulospora marina* I. Schmidt**

**Substrate**: Marine wood, *Phragmites*, and other plant remains.

**Distribution**: Germany, Mecklenburg-Vorpommern, Barther Oie, Kloster (Hiddensee) Seeseite, Kubitzer Bodden, Schaprode, Stralsund (Schmidt 1974, as *Vesicularia marina*).

*Note*. Originally described from the Baltic Sea as *Vesicularia marina* Schmidt (1974), but as this name was preoccupied, the new name *Cumulospora* was proposed (Schmidt 1985). The same fungus was also described by Abdullah et al. (1989) as *Basramyces marinus* from *Phragmites australis* (Syn. *Phragmites communis*) in southern marshes of Iraq. Recorded by Schmidt (1974; *V. marina*) as an obligate marine species occurring in the meso- to oligohaline zones. *Cumulospora marina* is distributed from temperate to tropical locations and often found on mangrove bark with collection from Egypt and Thailand (Abdel-Wahab et al. 2010).

**20. *Dichotomopilus indicus* (Corda) X.Wei Wang and Samson**

**Substrate**: Culms of *Ammophila*, and driftwood.

**New record**: Sweden. Gotland, Ardre par., Folhammar, 57°20ʹ45.03”N, 18°43ʹ59.11”E, ST19-10b (UPS). Fårö par., Ekeviken, 57°58ʹ30.28”N, 19°15ʹ24.69”E, ST19-78 (UPS). Fårö par., Ekeviken, 57°58ʹ30.28”N, 19°15ʹ24.69”E, ST19-30b (UPS). ST19-32b (UPS); Sudersand, 57°57ʹ8.06”N, 19°15ʹ24.69”E, ST19-122 (UPS).

*Note*. A rather omnivorous terrestrial species here recorded from a habitat usually considered “marine”, viz. *Ammophila*, and driftwood. Previously recorded (Eriksson 2014) from the warship *Vasa* that sunk in 1628 and salvaged in 1961.

**21. *Dictyosporium pelagicum* (Linder) G.C. Hughes ex E.B.G. Jones**

**Substrate**: Marine wood, *Phragmites*.

**Distribution**: Germany. Mecklenburg-Vorpommern, several localities (Schmidt 1974).

Sweden. Uppland (Tibell et al. 2019).

*Note*. Recorded by Schmidt (1974) as an obligate marine species occurring in the meso- to oligohaline zones. Many collections recorded from various locations in Denmark (Rees et al. 1979; Koch and Jones 1983). An oceanic species distributed in temperate waters of the Atlantic and Pacific oceans.

**22. *Digitatispora marina* Doguet**

**Substrate**: Marine wood.

**Distribution**: Sweden. Öland (Henningsson 1974).

*Note*. An oceanic species known from various localities of the temperate parts of the Atlantic and Pacific coasts (Kohlmeyer and Kohlmeyer 1979). Reported by Koch and Jones (1983) from Denmark and easily missed by its white resupinate fruit body.

**23. *Diplodia orae-maris* Linder**

**Substrate**: Driftwood.

**New records**: Sweden. Gotland, Fleringe par., 2 km NWE of Kapellshamn, Kapellshamnsviken, 57°52ʹ4.36”N, 18°48ʹ54.43”E, ST19-46, ST19-50 (UPS). Näs par., Nisseviken, 57°07′54″N, 18°13′02″E, ST19-20 (UPS). Södermanland, Trosa par., Askö, Storsand, 58°48′27″N, 17°41′07″E, ST19-111 (UPS).

*Note*. Originally described from the East Coast of the USA, this oceanic species has also been reported from the Atlantic coast of Europe. **New** to the Baltic Sea, and to Gotland.

**24. *Diplodia thalassia* N.J. Artemczuk**

**Substrate**: Driftwood.

**Distribution**: Sweden. Gotland, Näs par., Nisseviken, 57°07′54″N, 18°13′02″E, ST19-12 (UPS).

*Note*. Originally described from sediments of the Black Sea (Artemczuk 1980), this might be a species confined to brackish water. **New** to the Baltic Sea, and Sweden.

**25. *Emericellopsis maritima* Beliakova**

**Substrate**: Driftwood.

**Distribution**: Sweden. Gotland, Ardre par., Folhammar, 57°20ʹ45.03”N, 18°43ʹ59.11”E, ST 19-01d (UPS; GenBank MT072095).

*Note*. Originally described from the Black Sea (Belyakova 1970), this may well be a species confined to brackish water. Reported from Japan (Udea 1995). GenBank records for nuITS sequences (13 records, where four of them refer to sequences from the “type material of *Emericellopsis maritima*” and culture collection “CBS:491.71”) are verified from Canada, China, Ireland, and Poland. **New** to the Baltic Sea, and Sweden.

**26. *Halenospora varia* (Anastasiou) E.B.G. Jones**

**Substrate**: Driftwood.

**Distribution**: Germany, Mecklenburg-Vorpommern, several localities (Schmidt 1974b).

Sweden. Ångermanland and Gotland (Henningsson 1974 as *Zalerion varium*), Uppland (Tibell et al. 2019).

**Additional record**: Sweden. Gotland, Fårö par., Ekeviken, 57°58ʹ30.28”N, 19°15ʹ24.69”E, ST19-30a (UPS). Näs par., Nisseviken, 57°07′54″N, 18°13′02″E, ST19-60a (UPS). Uppland, Älvkarleby par., Rullsand, 60°38′28″N, 17°28′31″E, ST19-102 (UPS).

*Note*. Recorded by Schmidt (1974) as an obligate marine species occurring in the mesohaline zone. An oceanic species with a distribution in both the Atlantic and Pacific Oceans. **New** to Gotland.

**27. *Haligena elaterophora* Kohlm.**

**Substrate**: Marine wood.

**Distribution**: Sweden. Medelpad, Södermanland and Uppland (Henningsson 1974).

*Note*. Recorded by Schmidt (1974) as an obligate marine species occurring in the mesohaline zone. Reported by Rees et al. (1979) and Koch and Jones (1983) from various localities in Denmark. An oceanic species which has also been recorded from temperate coastal areas of the North Atlantic and the eastern Pacific Ocean.

**28. *Halosphaeriopsis mediosetigera* (Cribb and J.W. Cribb) T.W. Johnson**

**Substrate**: Marine wood.

**Distribution**: Germany. Mecklenburg-Vorpommern, several localities (Schmidt 1974a; 1974b; both as *Halosphaeria mediosetigera* and *Culcitalna achraspora*). Sweden. Bohuslän, Öland, Skåne, (Henningsson 1974, as *Culcitalna achraspora* ≡ *Trichocladium achrasporum* as a sexual morph). Uppland (Tibell et al. 2019).

##### Additional records

Gotland. Fårö par., Ekeviken, 57°58ʹ30.28”N, 19°15ʹ24.69”E, ST19-31 (UPS), ST19-103 (UPS), ST19-115 (UPS), ST19-119 (UPS).

*Note*. Described from Australia, it was recorded by Schmidt (1974) as an obligate marine species occurring in the meso- to oligohaline zones. An oceanic species with a distribution in tropical to temperate parts of the Atlantic, Indian, and Pacific Oceans. Rees et al. (1979) documented 23 collections on wood associated with sand from Blokhus, and Lǿkken, Jutland, Denmark. Also reported from Denmark by Koch (1974) and Koch and Jones (1983). The records from Södermanland and Uppland indicate a considerable tolerance to low salinity levels, hence here considered an euryhaline species. **New** to Gotland.

**29. *Halazoon fuscus* (I. Schmidt) Abdel-Wahab, K.L. Pang, Nagah., Abdel-Aziz, and E.B.G. Jones**

**Substrate**: Rhizomes and culms of *Phragmites*.

**Distribution**: Germany. Mecklenburg-Vorpommern, Darsser Boddenkette (Schmidt 1969–1985 as *Cirrenalia fusca*).

*Note*. Originally described from the Baltic Sea. Recorded by Schmidt (1974a; 1974b; *C. fusca*) as an obligate marine species occurring in the meso- to oligohaline zones. It has subsequently been recorded also from France and Japan (Abdel-Wahab et al. 2010). A halophilous species.

**30. *Halobyssothecium obiones* (P. Crouan *et* H. Crouan) Dayar., E.B.G. Jones, and K.D. Hyde**

**Substrate**: Marine wood, *Phragmites*.

**Distribution**: Germany. Mecklenburg-Vorpommern, Stralsund (Schmidt 1974 as *Leptosphaeria discors*). Sweden. Ångermanland, Uppland (Henningsson 1974, as *L. discors*).

*Note*. Recorded by Schmidt (1974b) as occurring in the mesohaline zone. Distributed in the Atlantic, Indian, and Pacific Oceans (Dayarathne et al. 2018).

**31. *Halojulella avicenniae* (Borse) Suetrong, K.D. Hyde, and E.B.G. Jones**

**Substrate**: Driftwood.

**New records**: Sweden. Gotland, Näs par., Nisseviken, 57°07′54″N, 18°13′02″E.

*Note*. An oceanic species originally described from a mangrove in India (see Ariyawansa et al. 2013), and also known from Australia, Malaysia, and Thailand. **New** to Europe, the Baltic Sea, and to Sweden.

**32. *Halokirschsteiniothelia maritima* (Linder) Boonmee and K.D. Hyde, in Boonmee, Ko Ko, Chukeatirote, Hyde, Chen, Cai, McKenzie, Jones, Kodsueb, and Bahkali**

**Substrate**: Marine wood.

**Distribution**: Germany. Mecklenburg-Vorpommern, Barth, Born, Kloster Boddenseite, Kubitzer Bodden, Warnemünde, Wissower Klinken (Schmidt 1974, as *Microthelia linderi*).

Sweden: Gotland (Tibell 2016). Skåne (Eriksson 2014).

**New record**: Sweden. Gotland, Ardre par., Folhammar, 57°20ʹ45.03”N, 18°43ʹ59.11”E, ST 19-07 (UPS).

*Note*. Recorded by Schmidt (1974) as an obligate marine species occurring in the mesohaline zone. Known from temperate parts of both the East and West Coasts of the North Atlantic Ocean. Also recorded on wood associated with sand from Blokhus, and Lǿkken, Jutland, Denmark (Rees and Jones 1985).

**33. *Halosphaeria appendiculata* Linder**

**Substrate**: Marine wood.

**Distribution**: Germany. Mecklenburg-Vorpommern, Binzer Bucht, Glowe, Karlshagen (Schmidt 1974).

*Note*. Recorded by Schmidt (1974) as an obligate marine species occurring in the mesohaline zone. Many records for Denmark (Koch 1974; Koch and Jones 1983). An oceanic species with distribution in subtropical to temperate areas of the Atlantic and Pacific Oceans.

**34. *Remispora hamata* (Höhnk) Kohlm. (**Figure 3(c))

**Substrate**: Marine wood and *Phragmites*.

**Distribution**: Germany. Mecklenburg-Vorpommern, Glowe, Karlshagen, Prohner Bach (Schmidt 1974).

*Note*. Recorded by Schmidt (1974) as an obligate marine species occurring in the meso- to oligohaline zones. An oceanic species with distribution in subtropical to temperate areas of the Atlantic and Pacific Oceans. This species was not recognised as a valid species by Jones et al. (2015) but our preliminary phylogenetic analysis of its 28 S rDNA suggests that this is a valid species but its taxonomic position remains to be determined (unpublished results).

**35. *Jalapriya toruloides* (Corda) D**’**souza, H.Y. Su, Z. Luo, and K.D. Hyde**

**Substrate**: Marine wood.

**Distribution**: Sweden. Öland, Södermanland (Henningsson 1974, as *Dictyosporium toruloides*).

*Note*. A terrestrial species with distribution in subtropical to temperate areas of both hemispheres, although seemingly more often reported from coastal localities. An earlier terrestrial record from leaves of *Ribes nigrum* has been reported in the Swedish Species Observation System (https://www.artportalen.se/inSwedish). Recorded from Denmark by Koch and Jones (1983). The record from Södermanland indicates a considerable tolerance to low salinity levels, hence here considered an euryhaline species.

**36. *Lautisporopsis circumvestita* (Kohlm.) E.B.G. Jones, Yusoff, and S.T. Moss**

**Substrate**: Driftwood.

**New records**: Sweden. Södermanland, Trosa par., Askö, Storsand, 58°48′27″N, 17°41′07″E, ST19-108 (UPS).

*Note*. Originally described from the west coast of North America, this species has also been recorded from Chile and North-western Europe. The record from Södermanland indicates a considerable tolerance to low salinity levels, hence here considered an euryhaline species. **New** to the Baltic Sea, and Södermanland.

**37. *Leptosphaeria australiensis* (Cribb and J.W. Cribb) G.C. Hughes**

**Substrate**: Driftwood.

**Distribution**: Sweden. Södermanland, Trosa par., Askö, Storsand, 58°48′27″N, 17°41′07″E, ST19-94 (UPS).

*Note*. Originally described from Australia, this oceanic species has proven to be spread in tropical to warm temperate parts of the Atlantic, the Indian, and Pacific Oceans. This is undoubtedly a species complex and further collections are required to sequence the marine *Leptosphaeria* species. **New** to the Baltic Sea, and Sweden.

**38**. ***Leptosphaeria albopunctata*** **(Westend.) Sacc.**

**Substrate**: Marine wood.

**Distribution**: Germany. Mecklenburg-Vorpommern, Bock, Stralsund (Schmidt 1974).

*Note*. Originally described from Belgium, this oceanic species has been recorded from temperate parts of coastal areas of both the eastern and western North Atlantic Ocean. **New** to Sweden.

**39. *Lignincola laevis* Höhnk**

**Substrate**: Marine wood.

**Distribution**: Germany. Mecklenburg-Vorpommern, Barthe, Darsser Bodenkette, Glowe, Grosse Jasmunder Bodden, Karlshagen (Schmidt 1974). Sweden. Gotland (Henningsson 1974; Tibell 2016), Skåne, Södermanland (Henningsson 1974), Småland (Tibell 2016).

**New record**: Sweden. Södermanland, Trosa par., Askö, Storsand, 58°48′27″N, 17°41′07″E, ST19-91 (UPS), ST19-96 (UPS).

*Note*. Originally described from the German North Sea coast, it was recorded by Schmidt (1974) as an obligate marine species occurring in the meso- to oligohaline zones. An oceanic species spread in tropical to temperate waters in both hemispheres, and occurs in the Atlantic, Indian, and Pacific Oceans.

**40. *Naïs inornata* Kohlm.**

**Substrate**: Marine wood.

**Distribution**: Germany. Mecklenburg-Vorpommern, Barther Oie, Kloster Boddensetie, Grosse Jasmunder Bodden, Stralsund (Schmidt 1974).

*Note*. Recorded by Schmidt (1974a, 1974b)) as an obligate marine species occurring in the meso- to oligohaline zones. Rees et al. (1979) record 44 collections on wood associated with sand from Blokhus, and Lǿkken, Jutland, Denmark. An oceanic species with a distribution in coastal, temperate areas of both the eastern and western North Atlantic and the Pacific Ocean.

**Figure 2.**
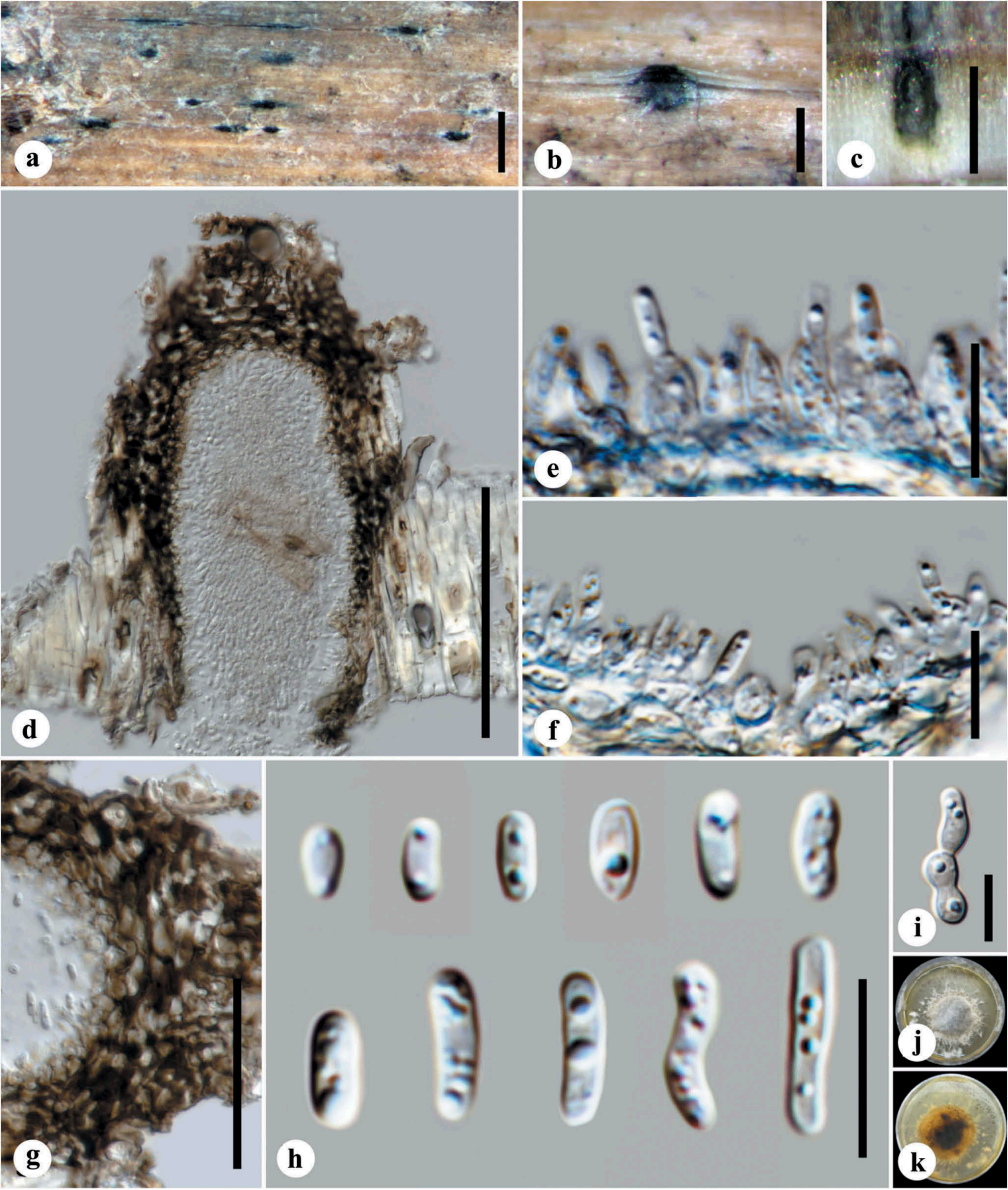
*Lentithecium lineare*: (A and B) Conidiomata on host surface. (C and D) Vertical sections of conidiomata. (E and F) Conidiogenous cells. (G) Peridium. (H) Conidia. (I) Germinating conidium. (J and K) Culture on malt extract agar (MEA, upper and lower sides, respectively). Scale bars: A = 1,000 µm, B and C = 200 µm, D = 100 µm, E and F = 10 µm, G = 50 µm, H and I = 10 µm.

**41. *Lentithecium lineare* (E. Müll. ex Dennis) K.D. Hyde, J. Fourn., and Ying Zhang (Figure 2**)

**Figure 3.**
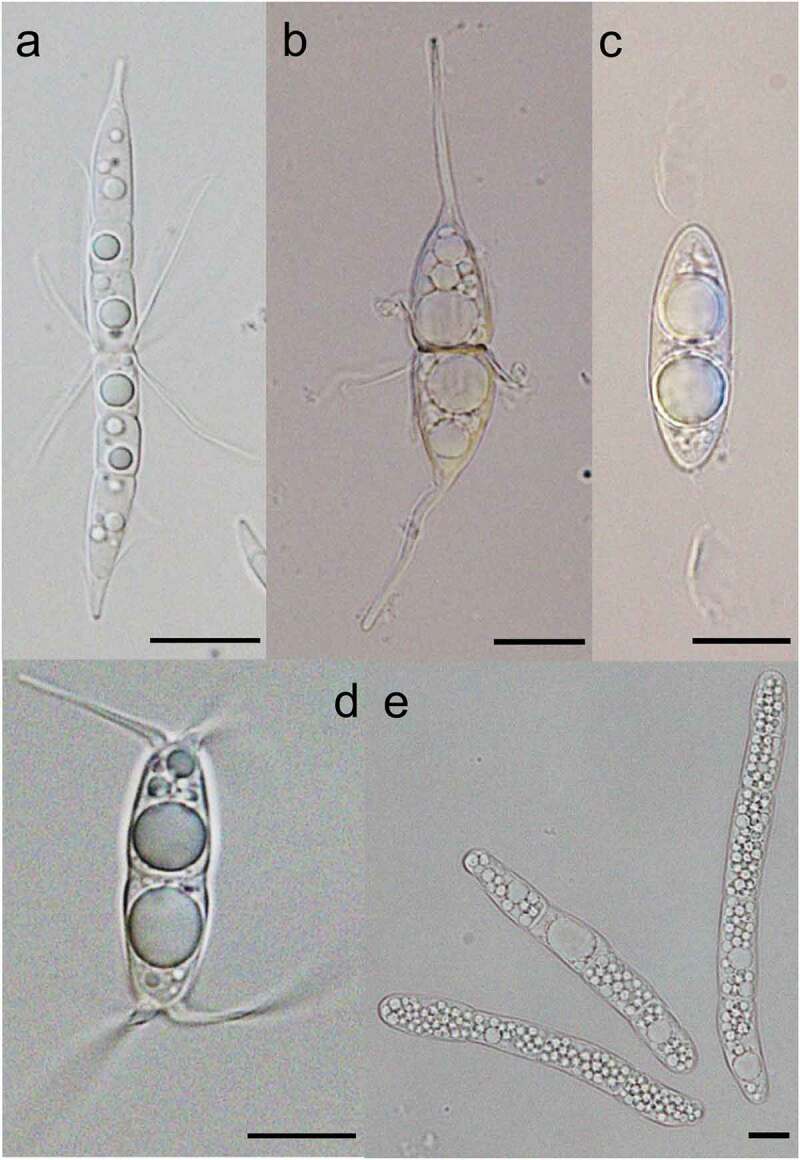
(A) *Corollospora quinqueseptata*, (B) *Corollospora gracilis*, (C) *Remispora hamata*, (D) *Arenariomyces trifurcatus*, and (E) *Setoseptoria phragmitis*. Scale bars: A = 30 µm, B–E = 10 µm.

**Substrate**: Driftwood.

**Distribution**: Sweden. Gotland, Fleringe par., 2 km NWE of Kapellshamn, Kapellshamnsviken, 57°52ʹ4.36”N, 18°48ʹ54.43”E, 03/07 2019 (EBG).

*Note*. This collection was identified as the asexual morph of *Lentithecium lineare* by sequence data and is illustrated in Figure 2. This is the first time an asexual morph has been described for this species. *Lentithecium* species are reported from both freshwater and marine habitats with *L. rarum* and *L. voraginesporum* listed in Jones et al. (2019), see website http://www.marinefungi.org. A new *Lentithecium* species will be described in follow-up paper.

*Note*. Originally described from Scotland, this species is known from *Phragmites* culms in Northwestern Europe. It was reported from the province of Skåne by Eriksson (2014) as *Keissleriella linearis* E. Müll.; the asexual morph.

**42. *Leptosphaeria pelagica* E.B.G. Jones**

**Substrate**: Marine wood.

**Distribution**: Sweden. Norrbotten and Uppland (both Henningsson 1974).

**New records**: Sweden. Gotland, Fårö par., Ekeviken, 57°58ʹ30.28”N, 19°15ʹ24.69”E, ST19-87 (UPS). Fleringe par., 2 km NWE of Kapellshamn, Kapellshamnsviken, 57°52ʹ4.36”N, 18°48ʹ54.43”E, ST19-46 (UPS). **New** to Gotland.

*Note*. Originally described on *Spartina* culms from England, this oceanic species has proven to have a distribution in coastal, temperate areas of both the eastern and western North Atlantic Ocean.

**43. *Leucosporidium scottii* Fell, Statzell, I.L. Hunter, and Phaff**

**Substrate**: Decaying algae.

**Distribution**: Sweden. Uppland (Tibell 2016).

*Note*. Originally described from Antarctica, this oceanic yeast species has a predilection for cold waters and has been recorded from several localities in the Atlantic, Indian, and Pacific Oceans (see https://www.gbif.org/search?q = Leucosporidium%20scottii).

**44. *Lizonia halophila* E. Bommer, M. Rousseau and Sacc., in Bommer and Rousseau**

**Substrate**: On *Honckenya peploides* on sandy shores.

**Distribution**: Sweden. Uppland, Sandhamn (Starbäck 1896).

*Note*. Originally described from the Belgian North Sea coast, this oceanic species is now known to have a distribution on the European Northwest Atlantic Ocean. Not listed as a marine species in Jones et al. (2019b). This is a little known ascomycete in the Pseudoperisporiaceae, and historically referred to many other genera including *Lizoniella, Mycosphaerella*, and *Sphaerulina*. Genus accepted in Hyde et al. (2013) but no cultures or sequences available for the type species. A halophilous species.

**45. *Lulwoana uniseptata* (Nakagiri) Kohlm., Volkm.-Kohlm., J. Campb., Spatafora, and Gräfenhan**

**Substrate**: Marine wood.

**Distribution**: Germany. Schleswig-Holstein, Schlei (Höhnk 1955 as *Helicoma maritima*). Mecklenburg-Vorpommern, Binzer Bucht, Kloster, Glowe, Neuendorf Boddenseite, Wissower Klinken (Schmidt 1974, as *Zalerion maritimum*). Sweden. Ångermanland (Henningsson 1974), Södermanland (Henningsson 1974).

*Notes. Zalerion maritima* was connected culturally and phylogenetically with the sexual morph *Lulwoana uniseptata* by Nakagiri (1984) and Campbell et al. (2005), respectively. Recorded by Schmidt (1974, as *Z. maritimum*) as an obligate marine species occurring in the mesohaline zone. Originally the sexual morph was described from Japan, this oceanic species is now known to have a distribution in the subtropical to temperate Eastern Pacific, and in the Eastern North Atlantic Oceans.

**46. *Lulworthia fucicola* G.K. Sutherl.**

**Substrate**: Marine wood.

**Distribution**: Germany. Mecklenburg-Vorpommern, Altefähr, Binzer Bucht, Hiddensee, Glowe, Grosse Jasmunder Bodden (Schmidt 1974).

*Note*. An oceanic species originally described from England on the brown seaweed *Fucus*, but reported and isolated from intertidal wood in Chile by Burgos and Shearer (1977) epitypified by Campbell (2005). Found also on *Spartina* culms in England (Jones, unpublished). Distributed also in Western and Eastern North Atlantic Oceans.

**47. *Lulworthia halima* (Diehl and Mounce) Cribb and J.W. Cribb**

**Substrate**: *Zostera*.

**Distribution**: Sweden. Småland, Södermanland (Eriksson 1982, as *Lulworthia maritima* fide Eriksson 2014).

*Note*. An oceanic species originally described from Canada, but distributed in temperate parts of both the Western Atlantic and the Pacific Oceans (Hawaii on palm driftwood https://www.gbif.org/occurrence/1,987,934,609).

**48. *Lulworthia medusa* (Ellis and Everh.) Cribb and J.W. Cribb**

**Substrate**: Marine wood.

**Distribution**: Sweden. Ångermanland (Henningsson 1974).

*Note*. An oceanic species originally described from the Atlantic coast of North America, but distributed in tropical to temperate parts of the Atlantic and Pacific Oceans (see https://www.gbif.org/occurrence/search?offset = 0&taxon_key = 2,565,847). Often found on *Spartina* culms.

**49. *Lulworthia opaca* (Linder) Cribb and J.W. Cribb**

**Substrate**: Marine wood.

**Distribution**: Germany. Schleswig-Holstein, Schlei (Höhnk 1955 as *Halophiobolus opacus*).

*Note*. An oceanic species originally described from the Atlantic coast of North America, but distributed in coastal, temperate parts of the eastern North Atlantic Ocean and also found in the eastern Pacific Ocean.

**50. *Magnisphaera spartinae* (E.B.G. Jones) J. Campb., J.L. Anderson, and Shearer**

**Substrate**: Marine wood. Generally, on *Spartina* spp.

**Distribution**: Germany. Mecklenburg-Vorpommern, Stralsund (Schmidt 1974, as *Haligena spartinae*). Sweden. Uppland (Henningsson 1974 as *Haligena spartinae*).

*Note*. Only recorded from a locality with very low salinity by Schmidt (1974b). Originally described from Britain, but has also been reported from the east coast of the USA. A halophilous species.

**51. *Neocamarosporium calvescens* (Fr. ex Desm.) Ariyaw. and K.D. Hyde, in Ariyawansa, Thambugala, Manamgoda, Jayawardena, Camporesi, and Saranyaphat**

**Substrate**: Driftwood.

**Distribution**: Sweden. Uppland (Tibell et al. 2019).

*Note*. Originally described from Sweden, this is a species mostly collected on land plants, but only recently (Tibell et al. 2019) observed from marine wood. Eight marine *Neocamarosporium* species are listed in the website http://www.marinefungi.org, often from salt marsh plants such as *Halimione portulacoides, Salicornia* spp., while *N. endophyticum* was described from *Zostera noltii*.

**52. *Nereiospora comata* (Kohlm.) E.B.G. Jones, R.G. Johnson, and S.T. Moss**

**Substrate**: Marine wood.

**Distribution**: Germany. Mecklenburg-Vorpommern, Bock, Darss, Grabower Bodden, Grosdse Jasmunder Bodden, Kloster, Lohme, Schaprode (Schmidt 1974). Sweden. Ångermanland, Södermanland, Uppland (Henningsson 1974, as *Corollospora comata*).

*Note*. Recorded by Schmidt (1974) as an obligate marine species occurring in the mesohaline zone. Originally described from the USA, this species has been recorded from coastal temperate areas of both the eastern and western parts of the North Atlantic Ocean.

**53. *Nereiospora cristata* (Kohlm.) E.B.G. Jones, R.G. Johnson, and S.T. Moss**

**Substrate**: Marine wood.

**Distribution**: Germany. Mecklenburg-Vorpommern, several localities (Schmidt 1974b).

Sweden. Uppland (Henningsson 1974, as *Corollospora cristata*; Tibell et al. 2019).

*Note*. Recorded by Schmidt (1974) as an obligate marine species occurring in the mesohaline zone. An oceanic species recorded from temperate parts of the eastern North Atlantic Ocean. Often reported as the asexual morph *Monodictys pelagica* (Mouzouras and Jones 1985).

**54. *Orbimyces spectabilis* Linder**

**Substrate**: Marine wood.

**Distribution**: Germany. Mecklenburg-Vorpommern, Gr. Jasmunder Bodden (Schmidt 1974). Sweden. Medelpad (Henningsson 1974).

*Note*. A rarely collected oceanic species known from temperate areas of both the eastern and western North Atlantic. Reported by Koch and Jones (1983) from Denmark.

**55. *Panorbis viscosus* (I. Schmidt) J. Campb., J.L. Anderson, and Shearer**

**Substrate**: Marine wood.

**Distribution**: Germany. Mecklenburg-Vorpommern, Barther Oie, Glowe, Kloster Seeseite (Schmidt 1974, as *Halosphaeria viscosa*).

*Note*. Originally described by Schmidt from the Baltic Sea as an obligate marine species occurring in the meso- to oligohaline zones. An oceanic species, but infrequent in tropical to temperate areas of the Atlantic and Pacific Oceans. Recorded from Japan by Abdel-Wahab et al. (2011).

**56. *Paradendryphiella arenariae* (Nicot) Woudenb. and Crous**

**Substrate**: In culture from driftwood. Generally with sand.

**Distribution**: Sweden. Gotland, Ardre par., Folhammar, 57°20ʹ45.03”N, 18°43ʹ59.11”E, ST19-01b (UPS).

*Note*. Described from France this oceanic species is distributed in subtropical to temperate waters of the Atlantic and the Pacific Oceans. **New** to the Baltic Sea, and Sweden.

**57. *Paradendryphiella salina* (G.K. Sutherl.) Woudenb. and Crous**

**Substrate**: Driftwood. Generally, on seaweeds or wood associated with sand.

**Distribution**: Germany. Mecklenburg-Vorpommern, Altefähr (Schmidt 1974, as *Dendryphiella salina*). Sweden. Skåne (Erneholm 1972 as *D. salina*).

**New records**: Sweden. Gotland, Ardre par., Folhammar, 57°20ʹ45.03”N, 18°43ʹ59.11”E, ST19-01c (UPS). Fårö par., Sudersand, 57°57ʹ8.06”N, 19°15ʹ24.69”E, GJ649 (Hb. EGB).

*Note*. Recorded by Schmidt (1974b) as a facultative marine species occurring in the mesohaline zone. Isolated from sand in Denmark by Rees et al. (1979). Described from Britain, this oceanic species is distributed in tropical to temperate waters of the Atlantic and the Pacific Oceans. **New** to Gotland.

**58. *Phaeosphaeria orae-maris* (Linder) Khashn. and Shearer**

**Substrate**: Marine wood.

**Distribution**: Germany. Mecklenburg-Vorpommern, Barther Oie, Stralsund (Schmidt 1974 as *Leptosphaeria oraemaris*). Sweden. Ångermanland, Södermanland (Henningsson 1974, as *L. orae-maris*). Uppland (Tibell et al. 2019). Bohuslän, Södermanland, Skåne, Uppland.

*Note*. This oceanic species was originally described from California but has later been shown to have a distribution in temperate areas of both the eastern and western North Atlantic Ocean.

**59. *Phaeosphaeria spartinicola* Leuchtm., in Leuchtmann and Newell**

**Substrate**: Driftwood.

**Distribution**: Sweden. Uppland (Tibell et al. 2019).

*Note*. Originally described from the USA, but now also known from the UK and Argentina.

**60. *Paraphaeosphaeria sporulosa* (W. Gams and Domsch) Verkley, Göker, and Stielow**

**Substrate**: Marine wood.

**Distribution**: Sweden. Gotland, GJ640 (Hb. EGB).

*Note*. Originally described as the asexual morph *Coniothyrium sporulosum* and a new record as a marine species, but previously known from freshwater in Korea (Goh et al. 2016). *Paraphaeosphaeria neglecta* is also known from aquatic habitats and isolated from a marine coral (http://www.marinefungi.org). **New** to the Baltic Sea, and Sweden.

**61. *Pleospora spartinae* (J. Webster and M.T. Lucas) Apinis *et* Chesters**

**Substrate**: Driftwood.

**Distribution**: Sweden. Uppland (Tibell et al. 2019).

*Note*. Originally described from *Spartina* sp. in the UK but now known also from temperate coastal areas of both the eastern and western North Atlantic Ocean.

**62. *Pleospora triglochinicola* J. Webster**

**Substrate**: Driftwood.

**Distribution**: Sweden. Västerbotten (Eriksson 2014), Uppland (Eriksson 2014; Tibell et al. 2019).

*Note*. Originally described from Norway, it is now known to have a distribution in temperate coastal waters of the eastern and western North Atlantic Ocean. Also known by its asexual morph *Stemphylium triglochinicola* (Shearer and Crane 1977).

**63. *Pseudeurotium zonatum* J.F.H. Beyma**

**Substrate**: Marine wood.

**Distribution**: Sweden. Öland (Henningsson 1974).

*Note*. Isolated from the warship *Vasa* that sunk in 1628 and was salvaged in 1961, *P. zonatum* is now known to have a distribution in temperate coastal waters of the eastern and western North Atlantic Ocean.

**64. *Pseudogymnoascus roseus* Raillo**

**Substrate**: Marine wood.

**Distribution**: Sweden. Öland (Henningsson 1974).

*Note*. A species originally described from soil but also associated with wood (Rice and Currah 2006), seems not otherwise to have been recorded from marine wood.

**65. *Remispora maritima* Linder**

**Substrate**: Marine wood.

**Distribution**: Germany. Mecklenburg-Vorpommern, numerous localities (Schmidt 1974a, 1974b as *Halosphaeria maritima*). Schleswig-Holstein, Schlei (Höhnk 1955, as *R. lobata* Höhnk nov. sp.). Sweden. Södermanland (Henningsson 1974 as *Halosphaeria maritima*).

**New record**: Sweden. Södermanland, Trosa par., Askö, Storsand, 58°48′27″N, 17°41′07″E, ST19-109 (UPS).

*Note*. Previously reported from Södermanland by Henningsson (1974; as *Halosphaeria maritima*). Recorded by Schmidt (1974b) as an obligate marine species occurring in the mesohaline zone. Rees et al. (1979) documented 23 collections on wood associated with sand from Blokhus, and Lǿkken, Jutland, Denmark. An oceanic species with a distribution in tropical to temperate areas of the Atlantic and Pacific Oceans.

**66. *Remispora pilleata* Kohlm.**

**Substrate**: Marine wood.

**Distribution**: Germany. Mecklenburg-Vorpommern, numerous localities (Schmidt 1974, as *Halosphaeria pilleata*). Sweden. Medelpad (Henningsson 1974), Södermanland (Henningsson 1974), Uppland (Henningsson 1974; Tibell et al. 2019).

*Note*. Recorded by Schmidt (1974b) as an obligate marine species occurring in the meso- to oligohaline zones. Rees et al. (1979) reported 68 collections on wood associated with sand from Blokhus, and Lǿkken, Jutland, Denmark. Originally described from the German Atlantic coast it is now known to be distributed in temperate to warm coastal waters of the eastern and western North Atlantic Ocean.

**67. *Remispora quadri-remis* (Höhnk) Kohlm.**

**Substrate**: Marine wood.

**Distribution**: Denmark. Sjaelland, Copenhagen (Höhnk 1955 as *Palomyces quadri-remis* Höhnk), also reported by Koch and Jones 1983). Germany. Mecklenburg-Vorpommern, numerous localities (Schmidt 1974a, 1974b as *Halosphaeria quadriremis*). Sweden. Ångermanland (Henningsson 1974 as *Halosphaeria quadri-remis*).

*Note*. Recorded by Schmidt (1974) as an obligate marine species occurring in the mesohaline zone. Originally described from the German Atlantic coast it is now known to be distributed in temperate to warm coastal waters of the eastern and western North Atlantic Ocean.

**68. *Remispora stellata* Kohlm.**

**Substrate**: Marine wood.

**Distribution**: Germany. Mecklenburg-Vorpommern, several localities (Schmidt 1974a, 1974b as *Halosphaeria stellata*). Sweden. Öland, Södermanland, Skåne, Uppland (all Henningsson 1974 as *H. stellata*).

*Note*. Recorded by Schmidt (1974b) as an obligate marine species occurring in the meso- to oligohaline zones. Rees et al. (1979) listed 30 collections on wood associated with sand from Blokhus, and Lǿkken, Jutland, Denmark. Originally described from the USA, this species has also been recorded from temperate areas of the eastern North Atlantic Ocean.

**69. *Saagaromyces glitra* (J.L. Crane and Shearer) K.L. Pang and E.B.G. Jones, in Pang, Vrijmoed, Kong, and Jones**

**Substrate**: Driftwood.

**Distribution**: Sweden. Uppland (Tibell et al. 2019).

*Note*. Originally described from Florida as *Nais glitra*, this species was reported from tropical locations (Jones and Pang 2012). Referred to *Saagaromyces* based on sequence data and unusual for the genus in lacking appendages (Pang et al. 2003).

**70. *Setoseptoria phragmitis* Quaedvl., Verkley, and Crous**

**Substrate**: *Phragmites* culm.

**New record**: Sweden. Södermanland, Trosa par., Askö, Storsand, on *Phragmites* culm buried in sand, 58°48′27″N, 17°41′07″E, ST19-98.

*Note*. The type material of this species was described from culms of *Phragmites australis* collected at Mai Po mangrove, Hong Kong, but whether the type was of a marine origin is unknown (Quaedvlieg et al. 2013). The water of the sandy beach at Askö, where this fungus was collected, is brackish. **New** to the Baltic Sea, and Sweden.

**71. *Sphaerulina orae-maris* Linder**

**Substrate**: Marine wood.

**Distribution**: Germany. Mecklenburg-Vorpommern, Kloster (Schmidt 1974). Sweden. Skåne (Eriksson 2014), Uppland (Tibell et al. 2019).

*Note*. Originally described from the USA, this species has a distribution in both the eastern and western North Atlantic Ocean. Further it has been recorded from the Pacific Ocean (California and Hawaii). Also reported from *Spartina* species (Jones 1963; Calado et al. 2015).

**72. *Stemphylium* cf. *maritimum* T.W. Johnson**

**Substrate**: Decaying algae, *Phragmites, Scirpus*, plant remains.

**Distribution**: Germany. Mecklenburg-Vorpommern, Altefähr, Barther Oie, Gr. Jasmunder Bodden, Kloster, Strahlsund (Schmidt 1974b).

*Note*. Recorded by Schmidt (1974b) as an obligate marine species occurring in the meso- to oligohaline zones. Originally described from the USA, this species has subsequently been recorded from the UK.

**73. *Toriella tubulifera* (Kohlm.) Sakay., K.L. Pang, and E.B.G. Jones**

**Substrate**: Marine wood.

**Distribution**: Germany. Mecklenburg-Vorpommern, Binzer Bucht, Karlshagen (Schmidt 1974b as *Halosphaeria* cf. *tubulifera*). Sweden. Öland, Skåne, Uppland (all Henningsson 1974 as *Ceriosporopsis tubulifera*).

*Note*. Originally described from the USA, this species has subsequently been shown to be distributed in temperate areas of both the eastern and western North Atlantic Ocean.

**74. *Trichocladium alopallonellum* (Meyers and R.T. Moore) Kohlm. and Volkm.-Kohlm.**

**Substrate**: Marine wood.

**Distribution**: Germany. Mecklenburg-Vorpommern, several localities (Schmidt 1967 and 1974 as *Humicola alopallonella*). Sweden. Norrbotten (Henningsson 1974 as *H. alopalonella*), Uppland (Tibell et al. 2019). Collected at Ekeviken 03/07/2019.

*Note*. Recorded by Schmidt (1974b) as an obligate marine species occurring in the meso- to oligohaline zones. Rees et al. (1979) isolated it at various depths in sand collected in Denmark and on wood baits. Originally described from North America, it has been shown to have a distribution in tropical to temperate areas of the Atlantic, Indian, and Pacific Oceans.

**75. *Trichocladium constrictum* I. Schmidt**

**Substrate**: Marine wood.

**Distribution**: Germany. Mecklenburg-Vorpommern, Barther Oie, Gross-Schwansee, Prohner Bach, Stralsund (Schmidt 1974, invalid; validly published Schmidt 1985).

*Note*. Originally described from the Baltic Sea, and recorded by Schmidt (1974) as an obligate marine species occurring in the meso- to oligohaline zones.

**76. *Trichocladium lignicola* I. Schmidt**

**Substrate**: Marine wood.

**Distribution**: Germany. Mecklenburg-Vorpommern, several localities (Schmidt 1974).

**New record**: Sweden. Fårö par., Ekeviken, 57°58ʹ30.28”N, 19°15ʹ24.69”E, ST19-118 (UPS).

*Note*. Originally described from the Baltic Sea and recorded by Schmidt (1974, invalid; validly published Schmidt 1985) as an obligate marine species occurring in the meso- to oligohaline zones. Few records of this species, needs to recollected and sequenced. **New** to Sweden.

**77. *Trichocladium melhae* E.B.G. Jones, Abdel-Wahab, and Vrijmoed**

**Substrate**: Driftwood.

**New record**: Sweden. Gotland. Ekeviken, 03/07/2019.

*Note*. Originally described from Hong-Kong (Jones et al. 2001), and also known from India, Japan, Malaysia, and Singapore, it was described as a tropical species. The disjunction to the Baltic Sea seems quite dramatic, but may probably just reflect the insufficient exploration of marine fungi at large. **New** to Europe, the Baltic Sea, and to Sweden.

## Discussion

The title of this paper includes the concept “marine fungi”, a concept that has been greatly discussed during the history of marine mycology. The recognition of “marine fungi” is particularly challenging in brackish waters, and its use might be claimed to be inappropriate or uninteresting. The distinction made here is based on the total distribution of each species. If a species has only been reported from oceanic waters, or clearly in most cases been found in the oceans (apart from in the Baltic Sea) it qualifies as marine. These assessments have also in part rested on the information in Schmidt (1974), where the occurrences of species in different salinity zones were indicated. After these elaborations and critical evaluation of earlier records, 77 species have been listed as marine and occurring in the Baltic Sea. These records encompass both records from the literature (first and foremost those of Höhnk, Henningson and Schmidt) and in addition several new discoveries from our field-work in the provinces of Gotland, and the Baltic shores of Södermanland and Uppland in Sweden. Most of the records were identified by their morphology, some by the sequencing of their nuITS, in which cases GenBank numbers have been provided (see also Supplement 1).

In addition to this, 18 species for reasons of nomenclatural deficiencies and/or unclear ecological preferences were excluded from the main list of marine fungi, including one new species for Sweden (not included in the main text, see Supplement 1). Several of these records are early and for various reasons hard to verify; others refer to terrestrial species considered of accidental or facultative occurrence in the Baltic.

The total distribution of each species has been briefly outlined, and most of the Baltic marine species have their main distribution in temperate waters of the Atlantic Ocean. *Corollospora luteola, Halojulella avicenniae*, and *Trichocladium melhae* are here recorded for the first time from Europe. Additionally *Corollospora gracilis, C. quinqueseptata, Diplodia thalassia, Emericellopsis maritima, Leptosphaeria australiensis, Paradendryphiella arenariae, Paraphaeosphaeria sporulosa*, and *Setoseptoria phragmitis* are for the first time reported from the Baltic Sea and Sweden. *Lautisporopsis circumvestita* and *Diplodia orae-maris* are new just for the Baltic Sea, while *Leptosphaeria albopunctata* and *Trichocladium lignicola* are new just to Sweden.

Interestingly some of the Baltic marine fungi, like *Halojulella avicenniae, Leptosphaeria australis, Setoseptoria phragmitis,* and *Trichocladium melhae* exhibit rather dramatic disjunctions to faraway tropical areas. *Diplodia thalassia* and *Emericellopsis maritima* have been described from the Black Sea and might be species confined to brackish waters. Two species, *Trichocladium constrictum* and *T. lignicola*, are presently only known from the Baltic Sea.

In conclusion, among the 77 species recorded only two belong in Basidiomycota, viz. *Digitatispora marina* and *Leucosporidium scottii*, while the vast majority belong to the Ascomycota. The most ascomyceteous speciose classes are Sordariomycetes (42) with most species in Microascales (29), Lulworthiales (6), and Sordariales (5); and Dothideomycetes (24) with Pleosporales (19) been the most speciose. In a metabarcoding study by Rojas-Jimenez et al. (2019), other phyla of fungi have also been recovered as sequences from the Baltic Sea including the Chytridiomycota (orders Rhizophydiales, Lobulomycetales, and Gromochytriales) and the Cryptomycota. Halosphaeriaceae is the family usually dominating in marine oceanic habitats, and the Baltic Sea (29). While many families are reported by one to two species, Chaetomiaceae (5), Leptosphaeriaceae (3), Lulworthiaceae (5), Phaeosphaeriaceae (3), and Pleosporaceae (5) harbour a few species. *Corollospora*, with six species, is the most species-rich genus in the Baltic.

## Supplementary Material

Supplemental Material
