# Supplementary material for: Marine fungi of the Baltic Sea
Source: Mycology. 2020 Mar 12;11(3):195–213. doi: 10.1080/21501203.2020.1729886 (PMC7534365; doi:10.1080/21501203.2020.1729886)
Supplement: Supplemental Material [file TMYC_A_1729886_SM7269.docx]

**Supplement 1**

**Records excluded from the list of Marine Fungi of the Baltic Sea for reasons of incomplete information, nomenclatural deficiencies and/or unclear ecological preferences.**

**1. *Alternaria alternata* (Fr.) Keissl**

**Substrate**: Marine wood, stems of *Potamogeton* and Characeae, rhizomes of *Phragmites*.

**Distribution**: Germany. Mecklenburg-Vorpommern, Ahlbeck, Barhöft, Barther Strom, Wissower Klinken (Schmidt 1974, as “*Alternaria tenuis*”).

*Note*. *Alternaria* species have often been reported from marine habitats, but it is doubtful if any of them is obligately marine (Jones et al. 2009). Recorded by Schmidt (1974) as a facultative marine species occurring in the meso- to oligohaline zones. A halophilous species.

**2. *Alternaria maritima* G.K. Sutherl.**

**Substrate**: Marine wood.

**Distribution**: Germany. Niedersachsen, Leuchtturm Hohe Weg (Höhnk 1955).

*Note*. *Alternaria* species have often been reported from marine habitats, but it is doubtful if any of them is obligately marine (Jones et al. 2009). A halophilous species.

**3. *Alternaria tenuissima* (Fr.) em. Neergaard**

**Substrate**: Marine wood and plant remains.

**Distribution**: Germany. Mecklenburg-Vorpommern, Ahlbeck, Darss, Gr. Jasmunder Bodden (Schmidt 1974).

*Note*. The identity of this record is not clear and the name applied by Schmidt may have resulted from a nomenclatural mistake. See also under *A. alternata.* Recorded by Schmidt (1974) as a facultative marine species occurring in the mesohaline zones. A halophilous species.

**4. *Alternaria* sp.**

**New record**: Sweden. Gotland, Näs par., Nisseviken, 57°07′54″N, 18°13′02″E, ST19-05a (UPS, GenBank MT072096).

*Note*. This record is based on a culture isolation. It closely matches an unidentified *Alternaria* (GenBank MK479285) obtained from ‘marine environment’ in Quatar, but at the same level of similarity and cover another eight sequences were located. These were referred to *Alternaria* spp. and *Lewia* spp. Three *Alternaria* isolations from Quatar were derived from marine water, whereas six *Lewia* isolations were obtained from *Salicornia* from Poland (5) and Japan (1) respectively.

**5**. ***Arthrobotrys cf. oligosporus*** **Fresen.**

**Substrate**: Marine wood, *Phragmites*.

**Distribution**: Germany. Mecklenburg-Vorpommern, Ahlbeck, Binzer Bucht, Glowe, Kloster, Stralsund, Warnemünde, Wissower Klinken (Schmidt 1974).

*Note*. This species is predatory on nematodes, and although this mode of nutrition is common in soil little is known about it in marine habitats although a handful of species have been reported from brackish waters and the sea. Recorded by Schmidt (1974) as a facultative marine species occurring in the mesohaline zones. A halophilous species.

**6. *Cadophora fastigiata* Lagerb. *et* Melin, in Lagerberg, Lundberg *et* Melin**

**Substrate**: Marine wood.

**Distribution**: Sweden. Ångermanland, Medelpad, Norrbotten, Öland, Södermanland (Henningsson 1974, as ”*Phialophora fastigiata*”).

*Note*. Originally described from terrestrial habitats in Sweden, but no known to have a wide distributed in cold to temperate areas of both hemispheres. Not obligately marine, but perhaps a facultative marine with limited survival in marine habitats.

**7*. Cosmospora butyri* (J.F.H. Beyma) Gräfenhan, Seifert & Schroers, in Gräfenhan, Schroers, Nirenberg & Seifert**

**Substrate**: Marine wood.

**Distribution**: Sweden. Norrbotten (Henningsson, 1974 as “*Acremonium butyri*”*.*

*Note*. Originally described from butter from Denmark there are no other records from aquatic habitats.

**8*. Dacrymyces macnabbii* D.A. Reid**

**Substrate**: Driftwood.

**Distribution**: Sweden. Gotland, Näs par., Nisseviken, 57°07′54″N, 18°13′02″E, ST19-59b (UPS).

*Note*. A terrestrial species adrift and tolerant of low salinity levels. Widely distributed in Europe.

**9*. Didymella glomerata* (Corda) Qian Chen & L. Cai**

**Substrate**: Marine wood.

**Distribution**: Sweden. Öland (Henningsson 1974 as “*Phoma glomerata*”).

*Note*. The identification may be questioned considering the multitude of species described in *Phoma* and the ensuing taxonomic and nomenclatural uncertainty. *Phoma glomerata* is a common parasite on land plants and seems to occur mainly in coastal areas.

**10*. Diplocladiella scalaroides*** **G. Arnaud ex M.B. Ellis**

**Substrate**: Pine bark.

**Distribution**: Germany. Mecklenburg-Vorpommern, Stralsund (Schmidt 1974).

*Note***.** Recorded by Schmidt (1974b) as a facultative marine species occurring in the mesohaline zone. A halophilous species. A widely distributed terrestrial species.

**11*. Epicoccum nigrum* Link**

**Substrate**: Marine wood, *Potamogeton*, *Chara.*

**Distribution**: Germany. Mecklenburg-Vorpommern, Barhöft, Gr. Jasmunder Bodden (Schmidt 1974).

*Note*. Recorded by Schmidt (1974b) as a facultative marine species occurring in the mesohaline zone. As a terrestrial species having an almost cosmopolitan distribution.

**12.** ***Fusarium culmorum*** **(Wm.G. Sm.) Sacc.**

**Substrate**: In culture from driftwood.

**Distribution**: Sweden. Gotland, Näs par., Nisseviken, 57°07′54″N, 18°13′02″E, ST19-16 (UPS, GenBank MT072099).

*Note*. This is a very widespread soil borne grass pathogen causing seedling blight. It has also been reported from *Ammophila*, a genus harbouring several fungal species considered marine.

**13*. Lentescospora submarina* Linder**

**Substrate**: Driftwood.

**Distribution**: Germany. Mecklenburg-Vorpommern, Binzer Bucht, Darss, Bock, Karlshagen, Neuendorf Boddenseite, Stralsund (Schmidt 1974a, 1974b).

*Note*. Recorded by Schmidt (1974) as an obligate marine species occurring in the mesohaline zone, and hence here considered an euryhaline species.

**14. *Patellaria atrata*** **(Hedw.) Fr.**

**Substrate**: Driftwood.

**New record**: Sweden. Gotland, Näs par., Nisseviken, 57°07′54″N, 18°13′02″E, ST19-41 (UPS). **New** to Gotland.

*Note*. A widely distributed terrestrial species occasionally reported from marine habitats.

**15*. Penicillium glaucoalbidum* (Desm.) Houbraken & Samson**

**Substrate**: Marine wood.

**Distribution**: Sweden. Södermanland (Henningsson 1974, as “*Thysanophora penicillioides*”).

*Note*. A species growing on conifer needle (Kendrick 1961) and not further reported from marine habitats.

**16. *Sarocladium strictum*** **(W. Gams) Summerb.**

**Substrate**: In culture from driftwood.

**New records:** Sweden: Gotland, Näs par., Nisseviken, 57°07′54″N, 18°13′02″E, ST19-04, ST19-11, ST19-11b (UPS, GenBank MT072097).

*Note*. Originally described from grass leaves from Germany this species has proven to be a widely distributed species occurring in soil, debris and as endophyte and parasite of plants and it may occasionally even infect immunocompromised humans.

**17*. Septonema hormiscium* Sacc.**

**Substrate**: Marine wood.

**Distribution**: Germany. Mecklenburg-Vorpommern, Aalbude, Anklamm, Gützkower Fähre, Demmin (Schmidt 1974b).

*Note*. Recorded by Schmidt (1974) as a facultative marine species occurring in the meso- to oligohaline zones. A widely distributed terrestrial plant parasite.

**18. *Trichothecium crotocinigenum*** **(Schol-Schwarz) Summerb., Seifert & Schroers**

**Substrate**: In culture from driftwood.

**New record**: Sweden. Fårö par., Ekeviken, 57°58'30.28"N, 19°15'24.69"E, ST19-37a (UPS), ST19-37b (UPS, GenBank MT072098).

*Note*. Originally described from *Trametes* fruit bodies from Hungary. A widely distributed species in Europe and also known from Australia. It contains the mycotoxin croticin. **New** to Sweden.
